# Performance of Excess Heat Factor Severity as a Global Heatwave Health Impact Index

**DOI:** 10.3390/ijerph15112494

**Published:** 2018-11-08

**Authors:** John Nairn, Bertram Ostendorf, Peng Bi

**Affiliations:** 1 School of Biological Sciences, The University of Adelaide, Adelaide, SA 5005, Australia; Bertram.Ostendorf@adelaide.edu.au; 2 Australian Bureau of Meteorology, Adelaide, SA 5000, Australia; 3 School of Public Health, The University of Adelaide, Adelaide, SA 5005, Australia; Peng.Bi@adelaide.edu.au

**Keywords:** heatwave intensity, heatwave severity, heatwave impact, heatwave index, heatwave event moments, early warning system

## Abstract

The establishment of an effective policy response to rising heatwave impacts is most effective when the history of heatwaves, their current impacts and future risks, are mapped by a common metric. In response meteorological agencies aim to develop seamless climate, forecast, and warning heat impact services, spanning all temporal and spatial scales. The ability to diagnose heatwave severity using the Excess Heat Factor (*EHF*) has allowed the Australian Bureau of Meteorology (the Bureau) to publicly release 7-day heatwave severity maps since 2014. National meteorological agencies in the UK and the United States are evaluating global 7-day and multi-week *EHF* heatwave severity probability forecasts, whilst the Bureau contributes to a Copernicus project to supply the health sector with global *EHF* severity heatwave projection scenarios. In an evaluation of impact skill within global forecast systems, *EHF* intensity and severity is reviewed as a predictor of human health impact, and extended using climate observations and human health data for sites around the globe. Heatwave intensity, determined by short and long-term temperature anomalies at each locality, is normalized to permit spatial analysis and inter-site comparison. Dimensionless heatwave event moments of peak severity and accumulated severity are shown to correlate with noteworthy events around the globe, offering new insights into current and future heatwave variability and vulnerability. The *EHF* severity metric permits the comparison of international heatwave events and their impacts, and is readily implemented within international heatwave early warning systems.

## 1. Introduction

There is an increasing need to refine policies to address climate change and future heatwave risks. Heatwave impacts to human health have been established as a global phenomenon [1]. Chronic heatwave impacts have been demonstrated in Australia, where a 2002 study [2] estimated 1000 people per year over the age of 65 die from heat related deaths. Numerous extreme events have resulted in the deaths of hundreds of people, which has led to the conclusion that heatwaves are Australia’s deadliest natural hazard [3]. Extreme heatwaves became internationally notorious following a 2003 European heatwave when France recorded 15,000 excess deaths [4]. Heatwaves extract a heavy toll upon vulnerable people and communities. Human health aspects of the very young, old aged, mental health, underlying disease and social disadvantage contribute to heatwave vulnerability. On rare occasions high intensity impacts spread to healthy people through failure of infrastructure, utilities, and inadequate adaptation strategies [5,6,7]. Heatwave trends and projections exhibit an increase in frequency and intensity under a warming climate [8,9,10,11,12,13], implying increased risks and the need for improved climatic extreme warning systems to reduce the risk of disasters [14,15].

Recent investigations have focused on the need to measure heatwave intensity in a manner that is meaningful for each location, yet seamless over broad spatial scales. Percentiles-based heatwave metrics have been recommended to satisfy the locality criteria [16], where an example of an intensity calculation that is meaningful to any sector is the Heat Wave Magnitude Index (HWMI) and its daily derivative HWMId [17]. Similar to HWMI, the Excess Heat Factor (*EHF*) [18] measures heatwave intensity at each location with an additional component to account for adaptation. Whilst similar in principle to HWMI, *EHF* has distinctions worthy of note. Rather than use maximum temperature alone, daily temperature is considered important due to minimum temperature compounding extremes through modification of the diurnal heating cycle [19,20].

Epidemiological studies [21,22,23,24,25,26,27] have demonstrated *EHF* severity dose/response skill for morbidity and mortality in Australia for both city and regional communities. These multidisciplinary studies have formed the basis for partnership discussions between health agencies, emergency services and the Bureau of Meteorology (the Bureau) for development of a national heatwave forecast and warning framework. International studies have also demonstrated *EHF*’s skill for epidemiological response [28] and mortality modelling [29]. *EHF* severity has been shown to be useful as an exposure index that scales well against human health impact for and between exposed locations but there is a lack of comparative studies to evaluate efficacy across different climates and broad spatial scales.

From an applied perspective, agencies tasked with generating the necessary environmental assessments, forecasts and warnings must consider how policy makers across health, infrastructure, utilities, and emergency services can prepare and adjust to future climate scenarios. Choice of heatwave indices suitable for use in these systems must satisfy the following criteria:

1. Extreme values match user experience,
2. Useful as an indicator of impact,
3. Seamless interpretation across climate records, 7-day, multi-week, seasonal and climate projection forecasts,
4. Ease of interpretation, and common to both policy and operational users
5. Mapped to provide timely and locally specific guidance, and
6. Operate within a multi-hazard warnings framework

National meteorological agencies in Australia, the UK and United States have either put into operation or under evaluation the Excess Heat Factor [18] for global heatwave severity analysis and forecasts. The Bureau’s heatwave service has published national 7-day heatwave severity maps on the internet since 2014 [30]. The UK Met Office is evaluating global 7-day probability maps of heatwave (and coldwave) severity within their Global Hazard Map (GHM) project [31] whilst the Bureau [32] and NOAA (personal communication, University of Maryland) have funded experimental multi-week heatwave severity probability forecasts. The Bureau is also contributing global *EHF* heatwave severity maps to the Copernicus project [33] for users to envisage meaningful heatwave climate change scenarios. In support of further development and adoption of *EHF* severity as an international heatwave impact metric, this study will assess its skill across different climates around the globe.

In this paper, we question how *EHF* severity is related to health impacts as a globally comparable, quantitative indicator. We are using extreme heatwave events for which impacts are well documented and locations for which long-term climate data are available with the aim to investigate their relationships with human health impacts using various health outcomes. The relationship between heatwave indicators and health impact is then compared between sites around the globe to understand if a common response to heatwave severity is detectable.

## 2. Materials and Methods 

Meteorological data sites have been selected based on the availability of impact data, geographical distribution across Europe, Asia, North America and Australia, and heatwave events that have been examined in the literature.

Maximum and minimum temperature data were accessed from the Australian Bureau of Meteorology, National Centers for Environmental Information (US, UK and Asia) and European Climate Assessment and Dataset [34]. Sites chosen are listed in Table 1, showing the period for which data was available at each site. Time-series site data were examined and treated to remove data gaps, with subsequent ranked severity moments checked for false events due to data gaps.

*EHF* heatwave intensity and severity moment calculations for all stations listed below have been tabulated and are available as Supplementary Materials in a spreadsheet.

Heatwave intensity and severity were calculated using the technique described by Nairn and Fawcett [18]. *EHF*’s assembly (Equation (1)) from long (Equation (2)) and short-term (Equation (3)) daily mean temperature (*DMT*) anomalies creates a power-law time series that permits a novel normalization technique to build a dimensionless severity index (Equation (4)).

(1) $$EHF=EHI_{sig}\cdot \max\left(1,EHI_{accl}\right)$$

(2) $$EHI_{sig}=DMT_{3-day}-DMT_{95}$$

(3) $$EHI_{accl}=DMT_{3-day}-DMT_{30-day}$$

(4) $$Severity=EHF÷EHF_{85}$$

The principals and full derivation of this technique are reviewed in Appendix A, supported by examples. *EHI_sig_* denotes significance of heat events and *EHI_accl_* quantifies heat events requiring an adaption or acclimatization response, respectively. *EHF* has units of [°C^2^_L_], with the non-SI unit subscript (L) used to indicate the locality constraint. As a percentile-based temperature anomaly heatwave intensity index, *EHF* values are unique for every location. For example, smaller anomalies are found in the tropics compared to the mid latitudes due to differences in the climatic temperature range. As a measure of impact in exposure/response studies these intensity values are only meaningful at each location. In order to create an exposure/response index that can be used for both temporal and spatial studies *EHF* severity has been developed (Equation (4), see Appendix A for full description). Extreme value theory, (points over threshold) has been used to normalize *EHF* into a dimensionless severity index. In addition to the ability to compare heatwave impact spatial characteristics, impact thresholds have been successfully demonstrated for severity classes [18,23,24,26,27]. This study will investigate whether these severity classes exhibit common impact thresholds.

Moments of average, mean, median and standard deviation (amongst others) are associated with statistical properties of populations, or samples of populations. Heatwave climate indices have been developed [9] and can be thought of as heatwave climate moments (HCM). The concept of heatwave event moments (HEM) is introduced here to help distinguish the utility of heatwave intensity and severity for examining heatwave event impact. Heatwave event moments of peak (highest value during the event), load (integrated values across the event), length (days) and mean load are investigated for their relationships with human health impacts using various health outcomes.

HEM are defined using the following equations:

(5) $$Peak=\underset{event}{\max}Severity$$

(6) $$Load=\overset{finish}{\underset{start}{\sum }}Severity$$

(7) Length=count(severityevent)

(8) Mean=Load/Length

‘Peak’ denotes the highest heatwave severity recorded in a heatwave event and ‘Load’ is the integration of heatwave severity values for the duration of a heatwave event. Length is the number of days exceeding a severity threshold.

These heatwave event moments are initially discussed using mortality and morbidity data for the 2009 extreme heatwave that impacted Adelaide and Melbourne. The data are reproduced from a previous study [35] (p. 21, 22).

Daily London 1981 to 2016 mortality data have been sourced from the Medical & Environmental Data: A Mashup Infrastructure (MEDMI) project, through arrangements with the UK Met Office. Excess mortality was derived by averaging the mortality for a two-month period, centered on the heatwave event in the prior year or year earlier, depending upon the absence of heatwave conditions. Under this criteria, average mortality was calculated in 1982 for 1983; in 1988 for 1989; and in 1992 for 1994, 1995, and 1996.

**Figure 1:**
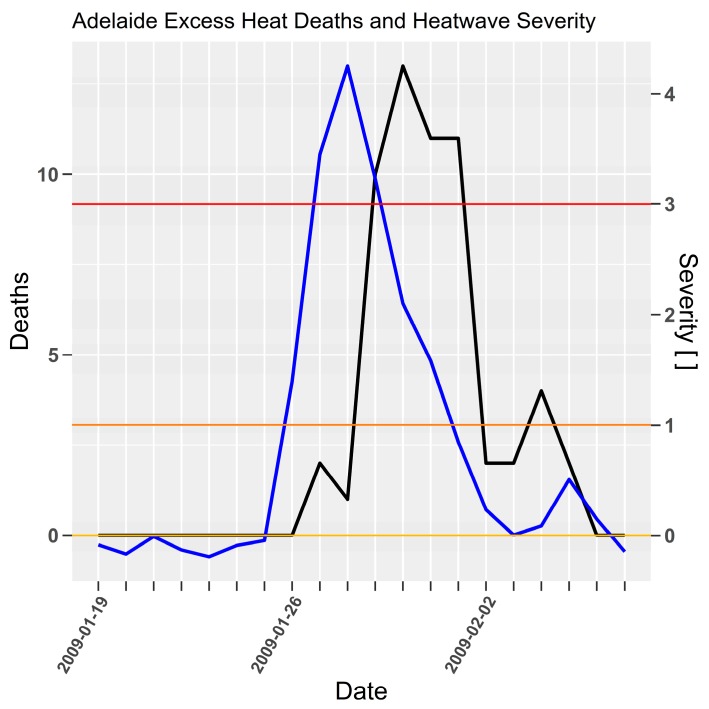
Heat related mortality (black line, left axis) and *EHF* severity (blue line, right axis) for Adelaide 2009 extreme heatwave [35] (p. 21).

Daily Chicago human health impact data have been sourced from publications [36,37,38] (p. 1516, Figure 1 and p. 1517, Figure 2; p. S159, Figure 1; and p. 174, Figure 1, respectively).

Daily Paris and Guangzhou human health impact data have been sourced from publications [39], (p. 1486, Figure 1) and [40] (p. 650 Figure 1).

A spreadsheet of all *EHF* severity moments for the cities listed in Table 1 is included in Supplementary Materials. Results for cities in bold are presented in results and discussed.

## 3. Results

### 3.1. Adelaide and Melbourne Heatwave Event Moments: Peak, Load, Length and Mean

Peak and load for both *EHF* intensity and severity are presented for Adelaide in Table 2. In this table Peak (Intensity) and Peak (Severity) are both valid impact measures, although the intensity is only meaningful for Adelaide (see Appendix A for explanation). Subsequent tables and discussion will only focus on the event severity moments as calculated in Equations (5)–(8).

Adelaide’s top peak and load moments range from 2.4 to 4.2 and 4.7 to 17.5, respectively, in Table 2. The 2009 event is top ranked for peak (4.2), load (17.5), and length (12). Mean (1.5) is strongly modulated by length and is not able to be interpreted in isolation from the other moments. As peak and load are considered to be superior moments, moments of length and mean are not always displayed in following tables but may be referenced in the Supplementary Materials spreadsheet.

A time-series of the 2009 event is shown in Figure 1, where heat related mortality lagged the rise and fall of severity by 2-days. As *EHF* under this formulation is designed as a lead indicator of heatwave impact the resultant lag suggests that the average heat accumulation over two or three days results in a scaled mortality response.

Melbourne’s extreme peak and load moments range from 2.7 to 6.1 and 3.8 to 15.7 respectively in Table 3. The 2009 event is ranked third for peak (4.9), second for load (14.2) and first for length (12, Supplementary Materials). The mean (1.2, Supplementary Materials) demonstrates the dependence upon load and length.

**Figure 2:**
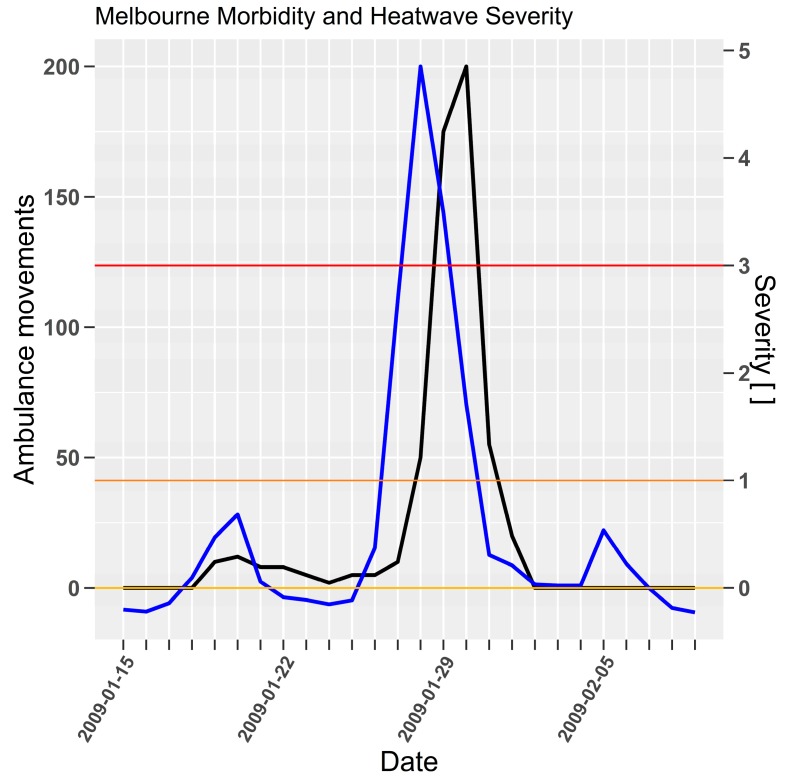
Heat related morbidity (ambulance movements, black line, left axis) and *EHF* severity (blue line, right axis) for Melbourne 2009 extreme heatwave [35] (p. 22).

Melbourne time-series for ambulance movements in Figure 2 responded to *EHF* severity in a similar manner to Adelaide’s mortality under the influence of similar HEM values.

In this case Adelaide and Melbourne human health impacts are linked strongly to peak and load moments, whilst the time-series shows severity leads impact by two days.

Coates et al. [3] (p. 41) tabulate other occasions when these cities may have been impacted by high impact heatwaves. Listed by State, recorded fatalities as shown in Table 4 have reasonable correlation with the heatwave event moments (HEM).

The spatial attributes of significant continental heatwave events may not affect Australia’s coastal capital cities each time a heatwave occurs in that state. Inland exposures are likely however, to have casualties taken to local major cities.

### 3.2. London Heatwave Event Moments: Peak, Load and Mortality

Hajat et.al. [41] (p. 370) heatwave study tabulated increases in deaths from 1976 to 1996. Table 5 includes these increases against HEM ranked by peak moment.

**Figure 3:**
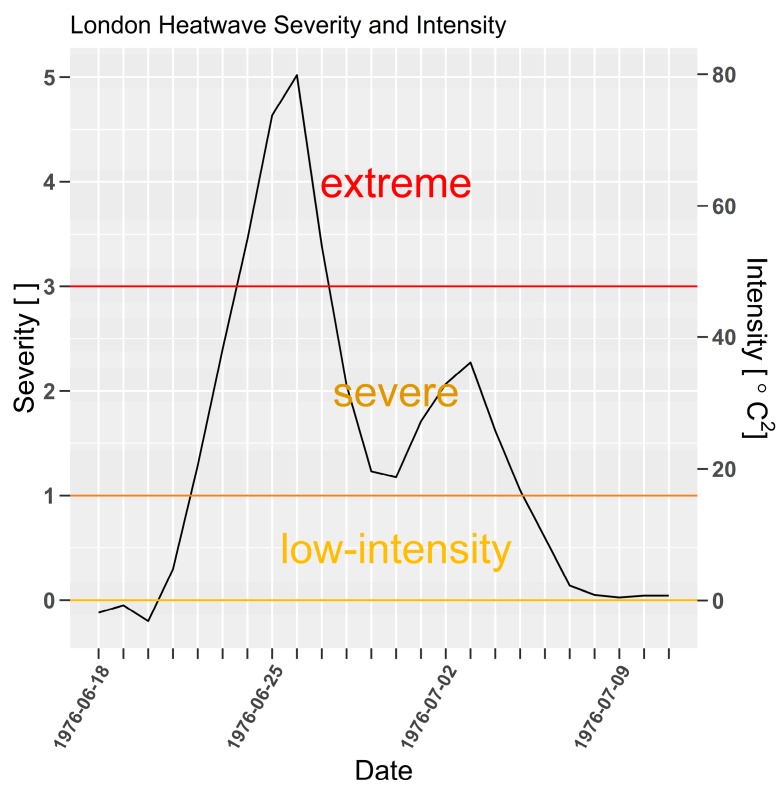
London (Heathrow) *EHF* severity and intensity for 1976 heatwave, calculated using site data. Dimensionless heatwave severity [ ] and intensity (*EHF*, [°C^2^]) on left and right *y*-axes respectively.

**Figure 4:**
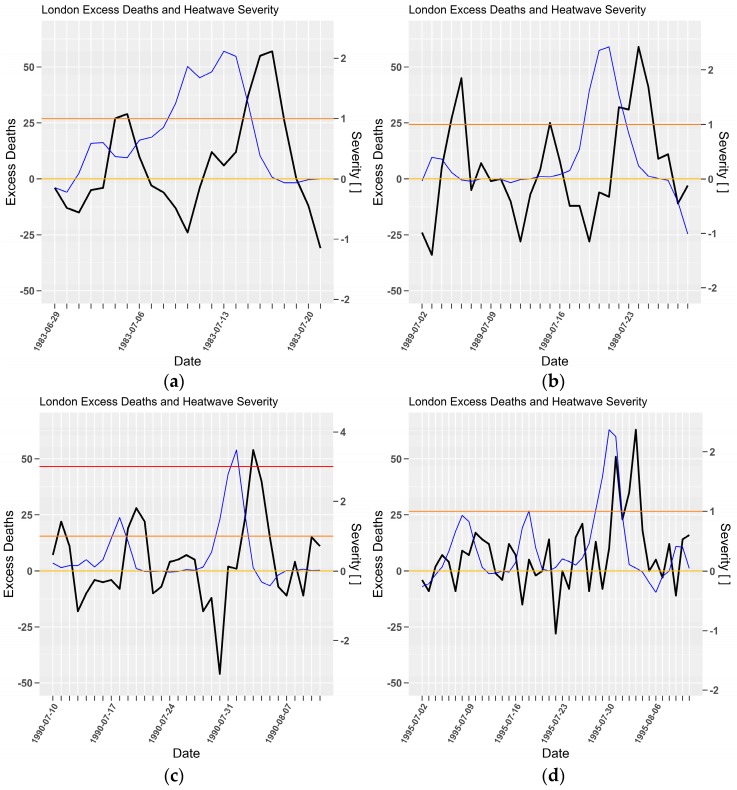
As per Figure 3. London (Heathrow) severity (blue) and mortality (black) for 1983 (**a**), 1989 (**b**), 1990 (**c**) and 1995 (**d**) heatwaves. Daily excess deaths and severity (dimensionless) on left and right *y*-axes, respectively.

**Figure 5:**
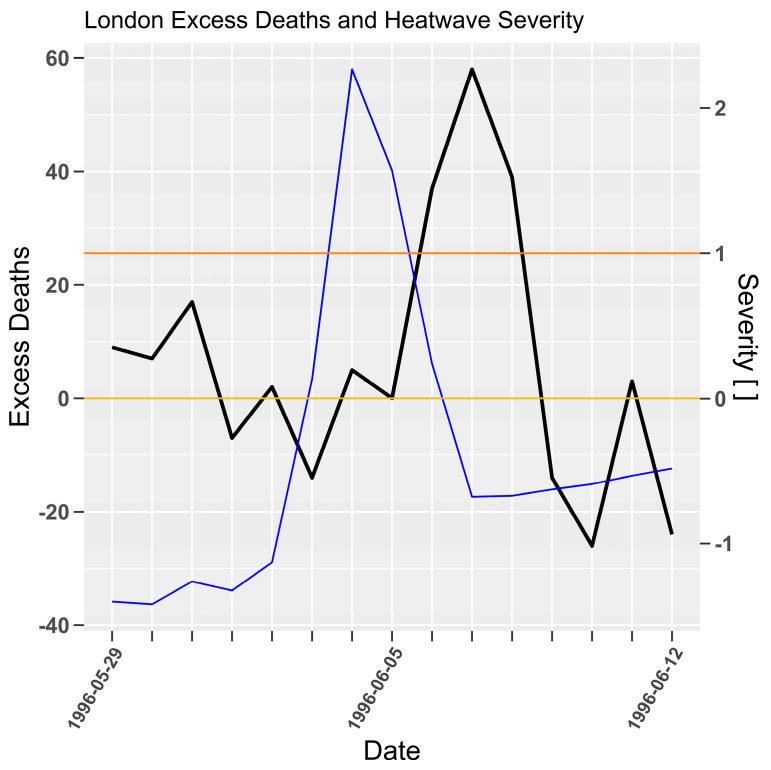
As per Figure 4. London (Heathrow) severity (blue) and mortality (black) for 1996 heatwave. Daily excess deaths and severity (dimensionless) on left and right *y*-axes respectively.

The heatwaves in 1976 and 1990 are ranked 1 and 2 for peak, and ranked 1 and 8 for load respectively. The event in 1976 which is also shown as a time-series in Figure 3, is substantially more intense than 1990 by all measures; Year (1976:1990), %Death (30.7:16.8), peak (5:3.5), load (34.6:10.2) and mean (1.6:1.1, Supplementary Materials). Whilst excess mortality data was unavailable for the 1976 heatwave, comparisons between Figure 3, Figure 4 and Figure 5 demonstrates the significance of this event. Only the 1990 event reached severity 3 compared to severity 5 in 1976 (site specific intensity is also shown in Figure 3). Table 5 indicates 1976 excess mortality would have been about two times larger than the 1990 event, and three times larger than the 1989 and 1983 events. Assuming a consistent exposure/response relationship, peak excess mortality of between 120 and 180 is likely to have occurred with a two to four-day lag to severity in 1976.

The heatwaves in 1983 and 1989 have similar peaks and mortality, although the load (16.6:9.9) is substantially higher in 1983. Figure 4a,b shows the 1983 event persisted longer (17:13, Supplementary Materials) with a higher mean (1.0:0.8, Supplementary Materials). All of the time-series events in Figure 4 show severity peak leads the mortality peak, usually by between two and four days. In all cases, where the intensity lingers near the severe threshold the mortality lags but appears to oscillate either side of the mean mortality, suggestive of a harvesting mechanism. Where the severity approaches and exceeds 2 there appears to be a strong, lagged pulse in excess mortality. In these cases, the recovery oscillation in mortality as the heatwave weakens does not appear to compensate for the mortality spike. The 1995 event recorded a temporary dip in excess mortality near severity 2, potentially indicative of interventions which were successfully protecting people. This appears to have been unsuccessful in subsequent days with excess mortality reestablishing a slightly delayed response to greater than severity 2.

In Table 5 the 1996 heatwave is unmatched with mortality data from the Hajat et al. [41] study. The heatwave severity and mortality time-series in Figure 5 once again shows a robust lagged mortality response for a severity moment of 2. It is unknown why the 1996 heatwave was not documented in the Hajat et al. study. However, excess mortality preceding the 1996 heatwave was elevated in a manner that was inconsistent with the 1983, 1989, 1990, and 1995 events. This may be an indicator of a separate adverse health event affecting the population that impacted epidemiological heatwave impact analysis. The response of about 25 excess deaths to severity 1 and about 60 excess deaths to severity 2 and over, holds for all events except 1990 where excess deaths were approximately 5 lower. This is a remarkably consistent response over a 14 year period whilst the corresponding average daily mortality rate fell from approximately 184 to 168 per day between 1982 and 1997.

For this observational sample (1921 to 2018) there are five other heatwaves that rank in the top 10 London events in Table 5. Notably, the 2003 event ranks second on load (17.4) and third on peak (3.1). The 2005 and 2017 events exhibit similar moment characteristics. More contemporary heatwaves (Supplementary Materials) in 2006, 2013, 2015, and 2016 have each reached the same peak (1.8) with variable load (13.0, 11.5, 5.0, 5.7). There were two events in 2006 where the second event was more significant (13.0 load) and longer (15, Supplementary Materials).

### 3.3. Chicago Heatwave Event Moments: Peak, Load

Chicago’s top four heatwaves rank peak and load in the same order (Table 6). The Chicago heatwave of 1995 is well documented as a devastating human health impact event [42]. The 1947 and 2012 events rank higher on both peak and load, however the 1995 length was shorter and returned a higher mean (Supplementary Materials). An investigation into the nature of the 1999 heatwave [42] noted the reduction in heatwave impact was not due solely to meteorological factors. Whilst the 14th ranked 1999 heatwave was still an intense event (Table 6) the lower peak of 2.2 and load of 5.1 shows it had significantly weaker meteorological heatwave severity moments than the 1995 heatwave.

**Figure 6:**
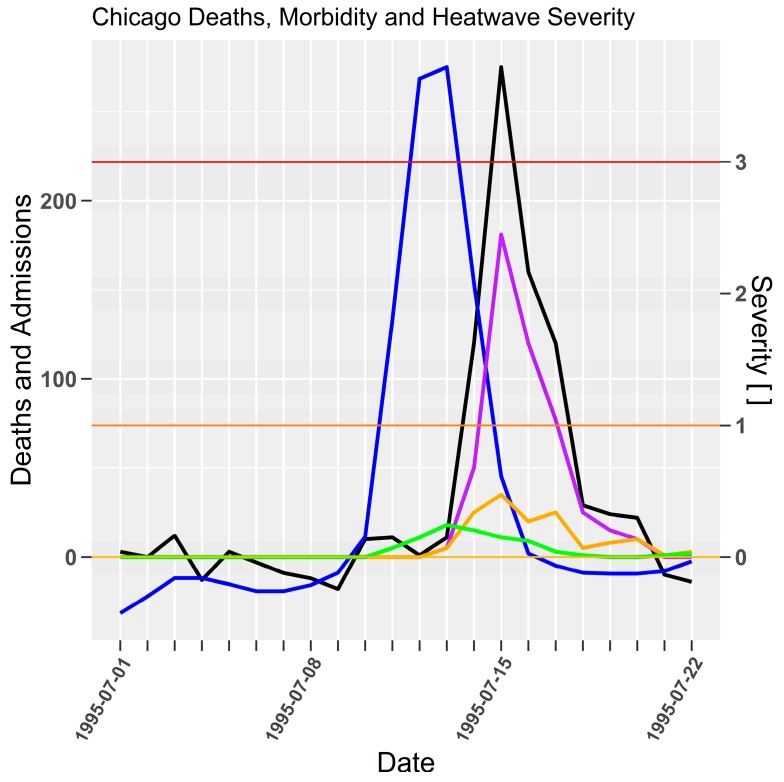
Chicago severity (blue), excess all cause deaths (black) (Whitman et al. [36] (p. 1517, Figure 2)), heat related mortality (purple) (Whitman et al. [36] (p. 1516, Figure 1)), heat deaths (gold) (Kaiser et al. [37] (p. S159, Figure 1)), and intensive care admissions (green) (Dematte et al. [38] (p. 174, Figure 1)) for 1995 heatwave. Daily deaths and admissions, and severity [ ] on left and right *y*-axes respectively.

The morbidity response for intensive care unit admissions shown in Figure 6 appears to be highly sensitive to severity due to the one-day lag. Mortality lag however, is consistent with prior examples at three to four-days. The three-day lag for excess all-cause, heat related and heat attributed deaths are coincident and show magnitudes consistent with each measure of mortality.

### 3.4. Paris Heatwave Event Moments: Peak, Load

The top three Paris heatwaves (2003, 1976, and 1948) in Table 7 have similar moments (peak, 3.5:3.4:3.3, mean, 1.7:2.0:1.7, Supplementary Materials), apart from the significantly lower load (31.2:35.2:11.9) in the 1948 event. The two top ranked heatwaves in 2003 and 1976 resulted in excess mortality across France of 15,000 and 6000 people respectively [43]. The difference in excess mortality for these two events can be attributed to changes in the vulnerability profile of the population or magnitude of the heatwave outside of Paris. Spatial analysis using gridded heatwave data would permit an accurate assessment of the change in vulnerability.

A heatwave study which modelled the expected mortality from the 2006 heatwave found an impact reduction attributed to improved intervention measures [43]. The 2006 peak (1.5), length and mean (30:0.5, Supplementary Materials) moments in Table 7 do not rank highly, although the load (15) ranks well (5). Any severity peak > 1 is considered to be a threat to vulnerable people and load was notable over the course of a lengthy heatwave. Each of these heatwave event moments may also assist in the assessment mortality model performance and assist in development of improved intervention measures.

**Figure 7:**
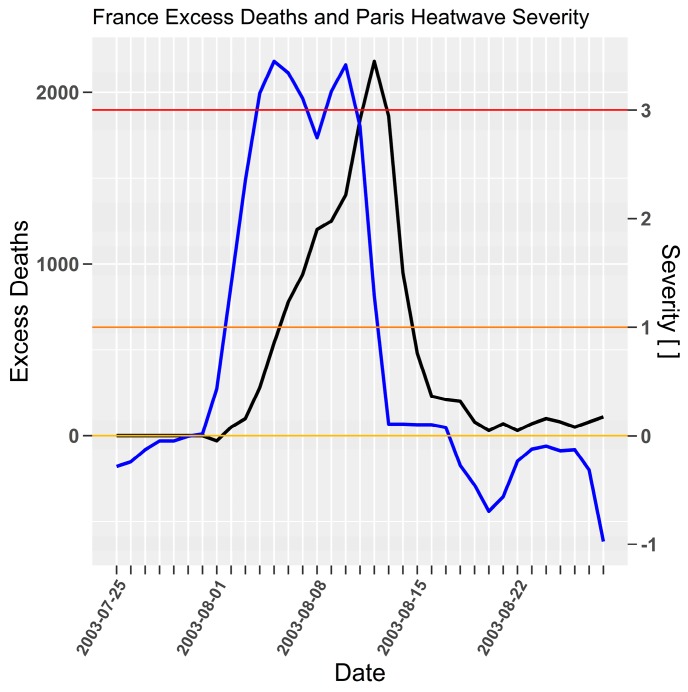
Paris severity (blue) and France excess mortality (black) for 2003 heatwave. Daily excess deaths (Poumadère et al. [39] (p. 1486, Figure 1)) and severity [ ] on left and right *y*-axes respectively.

**Figure 8:**
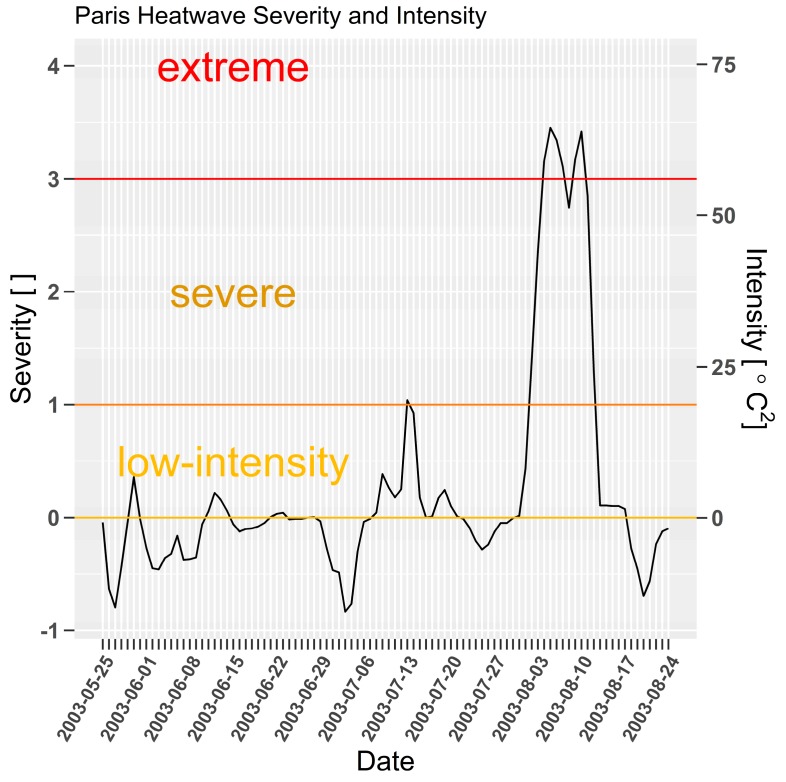
Paris (Orly) *EHF* severity and intensity for 2003 spring and summer, calculated using site data. Dimensionless heatwave severity [ ] and intensity (*EHF*, [°C^2^)] on left and right *y*-axes respectively.

The 2003 heatwave excess mortality lag in Figure 7 is unusual when compared to prior examples for Adelaide, London, and Chicago in that the growth in excess mortality is lagged by several days, yet falls with a familiar three-day lag. The delayed lag in this instance could be attributed to the effect of sustained low-intensity heatwaves beginning in late May 2003, and a brief severe heatwave during July, shown in Figure 8. Some improved adaptation measures may have developed during sustained pre-cursor low-intensity heatwaves until vulnerable people were overwhelmed by the prolonged extreme event. It is also difficult to compare excess mortality across France against a single station (Orly) as heatwave severity is unlikely to have evolved uniformly across the entire country.

### 3.5. Moscow Heatwave Event Moments: Peak, Load

A 2010 European heatwave resulted in 55,000 deaths across Russia [17]. Whilst the 2010 peak (2.2, Table 8) reached above the severe threshold (1) it was not extreme, and ranked 8 in the climate record. However, the load (46.5) is the highest found amongst the cities investigated in this study (Table 1). Virstu in Estonia (Supplementary Materials) recorded peak and load moments of 4.5, and 37.8 for this event, showing that Moscow was not the spatial locus for the extreme peak exposure.

The higher peaks (>3) recorded in 1958, 1996, 1998, and 2007 correlate with values where other cities have recorded high impact. Searches have not produced evidence of high impact for these events.

### 3.6. Guangzhou Heatwave Event Moments: Peak, Load

A central feature of Guangzhou’s top ranked heatwave severity is the lack of pre-21st century heatwaves in the top 20 (top 10 shown in Table 9). Whilst the 2005 heatwave ranked first on peak (5.5) moment, a recent 2018 event top ranked load (27.0). The recent development of more intense heatwaves supports observation of increased minimum temperatures compounding the intensity of heatwaves for southeast China [20].

**Figure 9:**
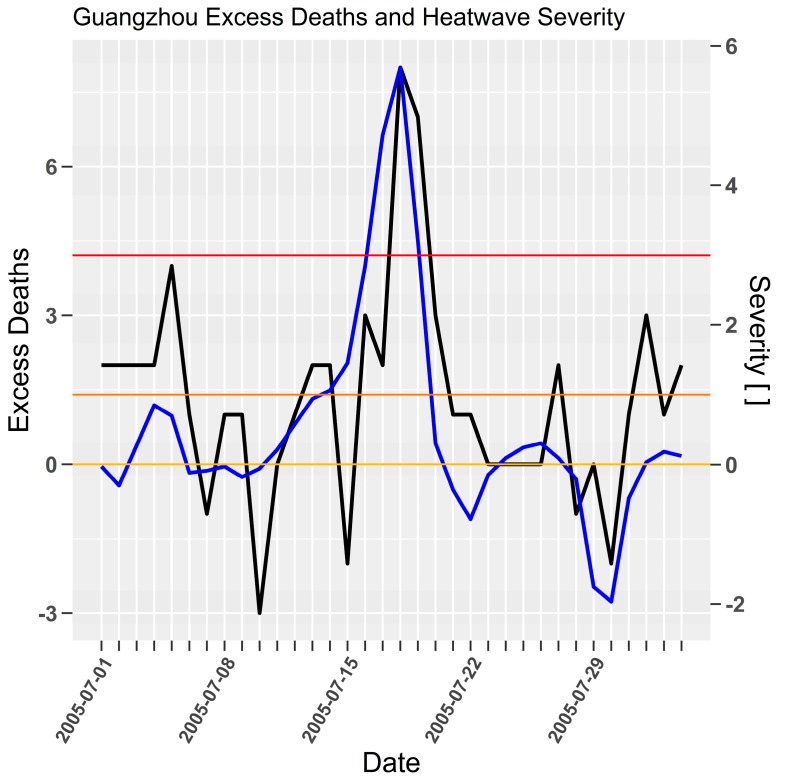
Guangzhou severity (blue) and excess mortality (black) for 2005 heatwave. Daily excess deaths (Jun Yang et al. [40] (p. 650, Figure 1)) and severity [ ] on left and right *y*-axes respectively.

The 2005 heatwave top ranked peak (5.5) and second ranked load (20.4). The time-series for excess mortality and severity in Figure 9 shows there is a noisier lag relationship between excess mortality and severity on this occasion. Excess mortality reaches a peak of 8 people on 8 July 2005 corresponding to severity >5. Whilst there appears to be a lack of power arising from the number of persons reported it is apparent that a one to two-day lag is present.

## 4. Discussion

*EHF* severity heatwave event moments (moments) of peak and load have been used to examine significant historical heatwaves. Ranking of these moments has placed these events in context with other significant exposure events.

The 1947 Chicago heatwave eclipsed the 1995 event (peak, 6.1:3.7 and load, 35.3:11.9). Changes in health record management and/or community resilience may have produced an impact record in 1947 less significant than the 1995 record. There are many examples in Section 3 and in the Supplementary Materials where top ranking heatwave severity events have occurred before high quality impact records commenced, highlighting the random nature of extreme events and the limits of quality impact records.

Spatial coherence of heatwaves is also revealed. The 2010 Russian heatwave might more correctly be called the 2010 Central European heatwave. Whilst Moscow top ranked load (46.5) moment for all events ranked in this study, Virstu, Berlin, Stockholm, Rome, Dresden, Nice and Ogulin all recorded higher peak moments (2.5–4.5, Supplementary Materials) compared to Moscow (2.2). This is a significant consideration following the time-series results for Adelaide, Melbourne, Heathrow, Chicago, Paris, and Guangzhou, where severity peaks greater than 2 have been shown to lead robust mortality and morbidity response.

Location and spread of heatwave impacts is also a significant phenomenon that will be addressed in following studies that will utilize gridded *EHF* data sets. A recent study into the impact of the 2015 European heatwave used *EHF* intensity moments to match and correctly rank heatwave exposure to health impact records in the Czech Republic [28]. Unable to access Czech temperature data for this study, analysis of nearby sites revealed that in 2015 Dresden recorded its top ranked peak and load heatwave event moments (4, 11.8, Supplementary Materials), with Munich, Berlin, Paris, Nice, Madrid, and Ogulin recording significant peak moments (2.3–3.0, Supplementary Materials). It would seem likely that many more excess deaths would have occurred outside the Czech Republic.

For some of the cities investigated in this study, a comparison of severity heatwave event moment impacts is not feasible due to the absence or variable nature of the impact data available. However, Adelaide, London, Chicago, and Guangzhou impacts are comparable assuming city domain impact data. Chicago 1995 excess deaths peaked at 275 persons for a severity peak of nearly 4. London excess death peaks ranged between 55 and 60 persons for severity peaks between 2 and 3, Adelaide excess deaths reached a peak of 13 persons for a corresponding severity peak over 4 whilst Guangzhou excess deaths peaked at 8 persons for a severity peak of nearly 6. There is little doubt that the 1995 Chicago event was a catastrophic impact event, which has been documented for societal compounding factors [44]. The stability of London impacts demonstrates an inherently more vulnerable and exposed environment when compared to Adelaide and Guangzhou. This is reinforced by comparison of the London 1976 and Guangzhou 2005 heatwaves where a peak severity of 5 in London resulted in a 30.7% increase in mortality whilst a peak severity of 6 in Guangzhou produced a 22% increase. Guangzhou appears to be more resilient than London to heatwaves. This result may be affected by under reporting of mortality given comparable populations and significantly different average mortality rates of these two cities.

Heatwave intensity and severity are repeatedly demonstrated as lead indicators for impact in the time series Figure 1, Figure 2, Figure 4, Figure 5, Figure 6, Figure 7 and Figure 9. Low-intensity heatwaves were associated with oscillating impacts that appeared to be consistent with a harvesting response, where initial rises in severity were followed by modest rises in impact, which then recovered below the long term mean mortality. This pattern was disrupted once the severity rose sharply to greater levels. At severity levels > 2 there was little evidence of a compensating impact recovery below the long term average mortality. Future epidemiological studies could consider whether a study period might be partitioned according to heatwave severity. This would reduce the statistical power for the extreme events given that they occur so rarely, but is consistent with the study of impacts arising from climate extremes.

Readers are encouraged to use the Supplementary Materials spreadsheet and query the data. Note: start and finish dates are numbered from 1 July for Australian, and 1 January for northern hemisphere stations.

## 5. Conclusions

The concept of heatwave event moments (HEM) has been introduced to improve the lexicon available to policy makers and responders. Until recently the wider community in Australia has relied upon heatwave length as a suitable measure for heatwave impact. Event severity peak and load have been shown to be superior indicators of impact.

The historical perspective of heatwave impact has been demonstrated by ranking severity peak and load. Heatwave severity moments have correctly ranked human health impact, especially when mortality was used as a health outcome. It is reasonable for policy makers to plan and implement mitigation measures in anticipation of predictable future heatwave severity impact.

Converting heatwave intensity into a dimensionless severity index permits comparison of impact scales between locations around the world. Common impact messages are readily constructed and communicated when good adaptive strategies are assumed for frequently occurring low-intensity heatwaves, whilst rarer more intense heatwaves have severe consequences for vulnerable people and even rarer and much more intense heatwaves produce extreme impacts if protective action is not undertaken. This work has demonstrated rising severity as a good lead indicator for increased human health impact. In the cases examined there is a noticeable shift in mortality mode as heatwaves become more intense. Lower severity is a good lead indicator, although the impact mortality response oscillates around the mean death rate, suggestive of harvesting cycles.

The ability to correctly scale impact events by ranked severity provides the community, response agencies, media, and policy makers with a common interpretive tool. Their experience of impact is validated and supported by the severity scale.

Heatwave event moments are common tools for historical and projection climate data that contextualize future scenarios against the lessons of the past. It is equally important that the same tools are visualized in seasonal and multi-week forecasts. The probabilistic nature of these products is helpful in preparing an appropriate mitigation strategy, particularly where likely exposure and impacts are well documented. Finally, when the same tools are used for short term forecasts and warnings, appropriate response levels can be initiated based upon a common language which has been in use across all spatial and temporal scales.

The statistical stability of *EHF_85_* has resulted in the application of heatwave severity on a continental scale for Australia’s 7-day heatwave service, evaluation within global probability severity maps in Australia, the UK and the US, and the creation of climate projection scenarios for the Copernicus project. The ongoing utility of these forecast trials is predicated on the effectiveness of *EHF* severity as a global impact metric.

The results presented in this paper support current national and international service developments based on peak severity heatwave event moment. There is some evidence that the severity load heatwave event moment may be required within an operational heatwave service, particularly for longer events.

Future research will focus on spatial climate records to further develop *EHF* severity climatic regimes and trends. Additional health outcomes indicators such as ambulance call outs, emergency department visits and hospitalizations will also be included in future assessments. These analyses will underpin interpretation of global *EHF* severity climate projection data generated for all impacted sectors, most notably health.

## Figures and Tables

**Table 1:** Excess Heat Factor (*EHF*) intensity and severity calculated for the observation period shown in grey bars for each site. Cities in bold are examined in detail in Section 3.

City	1850					1900					1950					2000		
**London** (Heathrow)																		
Madrid																		
**Paris** (Orly)																		
Nice																		
Munich																		
Dresden																		
Berlin																		
Virstu																		
Ogulin																		
**Moscow**																		
**Guangzhou**																		
**Melbourne**																		
Sydney																		
**Adelaide**																		
**Chicago** (O’Hare)																		

**Table 2:** Adelaide heatwave event peak, load, length and mean, using intensity and severity (1887 to 2018). Top 10 ranked for event peak. Peak and Load Intensity in units of [°C^2^]. Peak, Load and Mean Severity are dimensionless [ ] and Length in days.

Heatwave Period	Peak (Int)	Peak (Sev)	Load (Int)	Load (Sev)	Length	Mean (Sev)
26 January–6 February 2009	153	4.2	641.8	17.5	12	1.5
18–21 January 1875	121	3.3	418.6	11.4	7	1.6
11–17 January 2014	106.1	2.9	368.7	10.1	7	1.4
30 December 1899–2 January 1900	103.8	2.8	266.8	7.3	4	1.8
5–8 January 1930	94.9	2.6	173.7	4.7	4	1.2
25–29 December 1897	94.4	2.6	296.3	8.1	5	1.6
17–21 January 2006	93.7	2.6	209.4	5.7	5	1.1
17–23 January 1973	92.8	2.5	303.4	8.3	7	1.2
16–23 January 1982	90.5	2.5	367.7	10	8	1.3
8–13 January 1927	89.2	2.4	369.7	10.1	6	1.7

**Table 3:** Heatwave event peak and load using severity for Melbourne (1855 to 2018). Top 10 ranked for event peak severity. Severity is dimensionless [ ].

Heatwave Period	Peak	Rank	Load	Rank
12–17 January 2014	6.1	1	15.7	1
18–21 January 1875	5.2	2	13.2	4
26 January–6 February 2009	4.9	3	14.2	2
15–19 January 1959	3.7	4	8.4	7
14–20 January 1908	3.2	5	13.8	3
9–13 January 1905	3	6	7.5	
22–24 December 1920	2.9	7	4.7	
9–11 December 1998	2.8	8	3.8	
29 January–3 February 1912	2.7	9	8.3	8
18–22 January 2006	2.7	10	5.5	

**Table 4:** Heat Total Deaths for significant heat events in Australia, 1844–2011 [3] (p. 41) by State (WA, Western Australia; SA, South Australia; Vic, Victoria; NSW, New South Wales; Qld, Queensland) or City affected and heatwave event moments for affected city.

Date of Event	State or City Affected	Total Deaths	Heatwave Event Moments (City)
			Peak, Load, Mean, Length [ ], [ ], [ ], Days
January–February 1879	NSW, Vic	22	
October 1895–January 1896	WA, SA, Vic, Qld, NSW	435	5.6, 13.0, 2.6, 5 (Sydney)
January 1906	NSW, SA	28	
January 1908	Vic, SA, NSW	213	3.2, 13.8, 2.0, 7 (Melbourne)
January 1939	NSW, Vic, SA	420	2.4, 10.4, 1.5, 7 (Adelaide)
January–February 1959	Melbourne (Vic)	145	3.7, 8.4, 1.7, 5
January–February 2009	Vic, SA	432	Table 1 and Table 2

**Table 5:** Top ten ranked peak [ ] and load [ ] for London (Heathrow Airport, 1921 to 2018). Percentage change in deaths associated with heatwave period [42,43] (p. 370). # no heatwave was identified by Hajat et.al. [41] (p. 370).

Heatwave Period	%Death	Peak	Rank	Load	Rank
22 June–13 July 1976	30.7	5	1	34.6	1
28 July–5 August 1990	16.8 (5.4)	3.5	2	10.2	8
3–14 August 2003	-	3.1	3	17.4	2
15–21 June 2017	-	2.5	4	9.4	12
17–24 June 2005	-	2.5	5	10.1	9
16–28 July 1989	11	2.4	6	9.9	10
24 July–5 August 1995	7.1 to −0.3	2.4	7	9.5	11
5–8 June 1996	#	2.3	8	4.2	0
26 June–2 July 1952	-	2.2	9	7.4	14
3–19 July 1983	11.3 to 4.5	2.1	10	16.6	3

**Table 6:** Heatwave event peak and load using severity for Chicago (O’Hare Airport, 1946 to 2018). Top 10 ranked for event peak severity. Rank 14 inserted. Severity is dimensionless [ ].

Heatwave Period	Peak	Rank	Load	Rank
2–24 August 1947	6.1	1	35.3	1
28 June–8 July 2012	4.1	2	16.9	2
11–17 July 1995	3.7	3	11.9	3
19–22 June 1988	2.7	4	6.4	4
3–7 July 1977	2.7	5	6.8	10
29 June–2 July 1970	2.6	6	5.7	
28 July–5 August 1988	2.6	7	9.3	4
14–19 June 1994	2.6	8	8.2	
25 July–3 August 2006	2.4	9	8.3	7
22–26 June 2009	2.4	10	6	
2–6 July 1999	2.2	14	5.1	

**Table 7:** Heatwave event peak and load using severity for Paris (Orly Airport, 1921 to 2018). Top 10 ranked for event peak severity. 27th rank inserted. Severity is dimensionless [ ].

Heatwave Period	Peak	Rank	Load	Rank
1–18 August 2003	3.5	1	31.2	2
22 June–9 July 1976	3.4	2	35.2	1
26 July–1 August 1948	3.3	3	11.9	9
27 July–5 August 1990	3	4	11.5	11
22–30 July 1947	2.8	5	15.8	3
11–30 April 1921	2.8	6	15.4	4
25–28 June 1947	2.8	7	5.8	0
26 June–6 July 2015	2.8	8	11.2	0
29 June–7 July 1957	2.7	9	12.4	0
5–15 July 1923	2.7	10	11.6	10
8-28 July 2006	1.5	27	15.0	5

**Table 8:** Heatwave event peak and load using severity for Moscow (1948 to 2018). Top 10 ranked for event peak severity. Severity is dimensionless [ ].

Heatwave Period	Peak	Rank	Load	Rank
26–31 May 2007	4.3	1	16.1	4
7–14 July 1996	4.3	2	13.9	6
8–21 June 1998	3.7	3	20.5	2
25–29 May 1958	3.1	4	8.9	0
4–8 June 1988	2.6	5	6.1	0
9–18 July 1951	2.5	6	11.6	0
24 June–3 July 1991	2.4	7	9.4	0
1 July–18 August 2010	2.2	8	46.5	1
3–15 July 1954	2.1	9	10.5	0
16–20 July 1970	2	10	6.3	0

**Table 9:** Heatwave event peak and load using severity for Guangzhou (1945 to 2018). Top 10 ranked for event peak severity. Severity is dimensionless [ ].

Heatwave Period	Peak	Rank	Load	Rank
12–21 July 2005	5.5	1	20.4	2
26 July–1 Aug 2017	5.2	2	18.1	5
26 June–3 July 2004	4.4	3	16.1	6
23–30 July 2008	4	4	18.1	4
17–31 May 2018	3.5	5	27	1
13–17 June 2014	3	6	6.9	15
31 May–2 June 2014	2.8	7	3.6	0
7–9 July 2016	2.8	8	5.2	0
1–14 July 2010	2.7	9	20.2	3
31 May–2 June 2016	2.6	10	5	0

**Figure A1:**
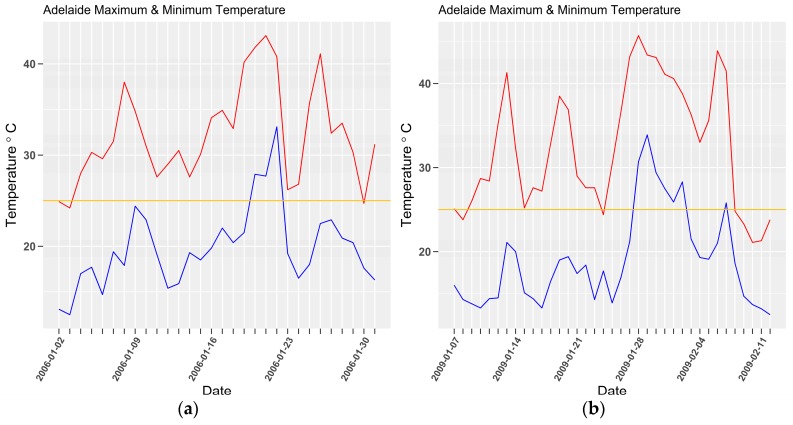
Daily maximum and minimum temperatures for Adelaide heatwaves in 2006 (**a**) and 2009 (**b**). 25 °C reference temperature (yellow line).

**Figure A2:**
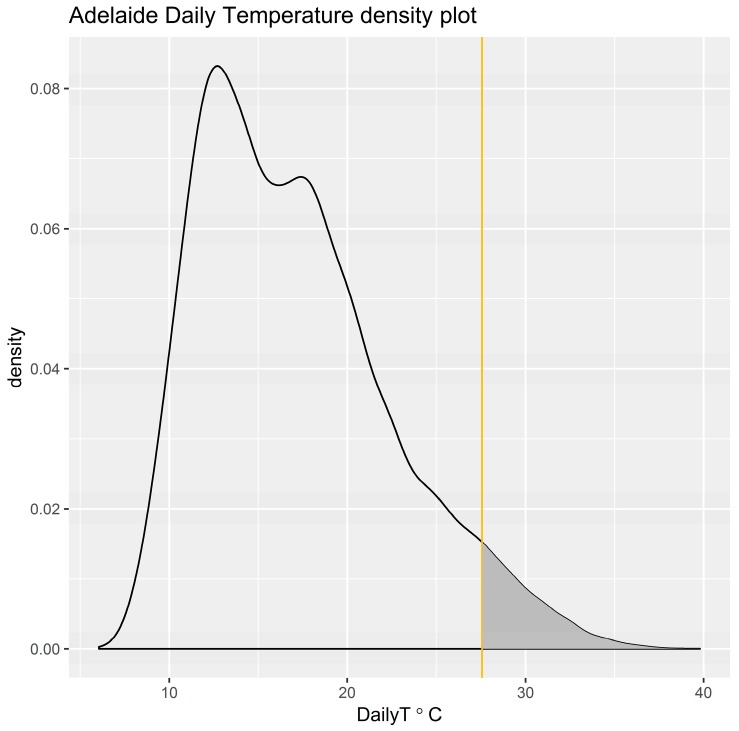
Distribution function of daily temperature for all days in 1971 to 2000 climate reference period. Grey shade shows 95th percentile tail for all heatwaves present for this reference period.

**Figure A3:**
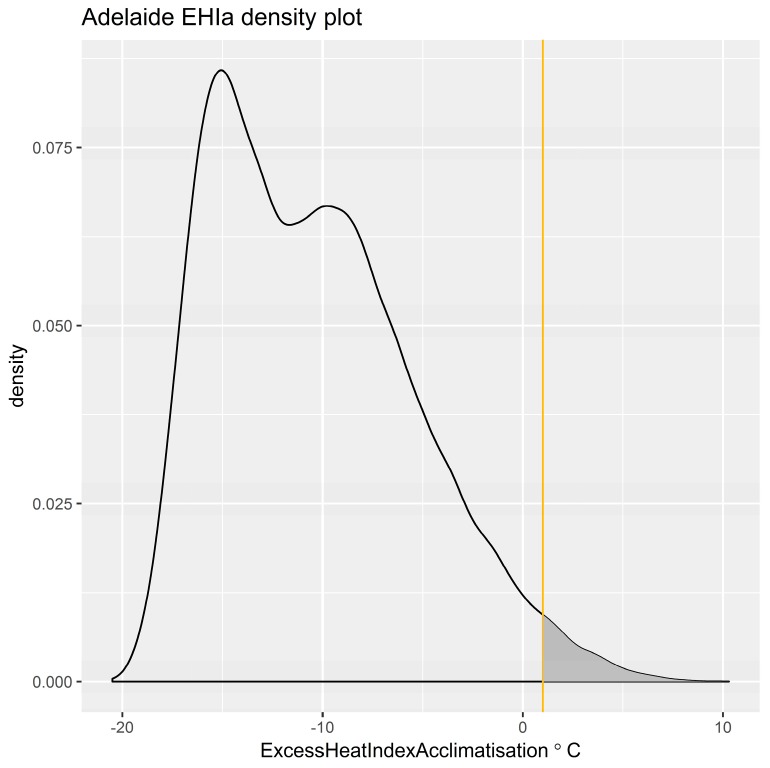
Distribution function of *EHI_accl_* for 1960 to 2018 climate period. Shaded region >1 shows acclimatization distribution samples when calculating *EHF*.

**Figure A4:**
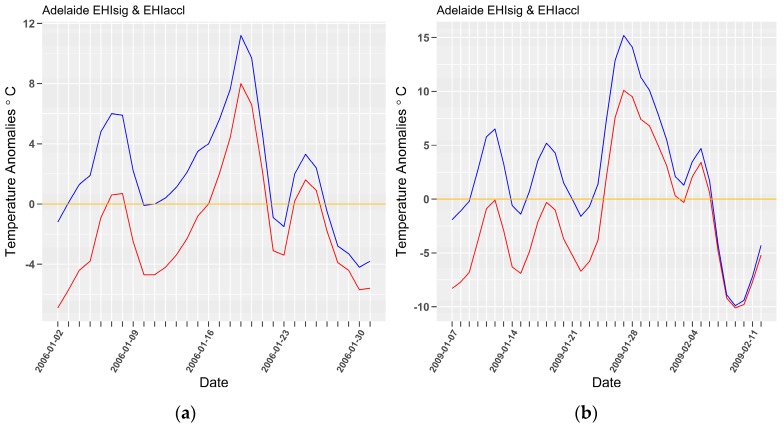
Acclimatization (blue) and significance (red) Excess Heat Indices for Adelaide’s 2006 (**a**) and 2009 (**b**) heatwaves, calculated using site data.

**Figure A5:**
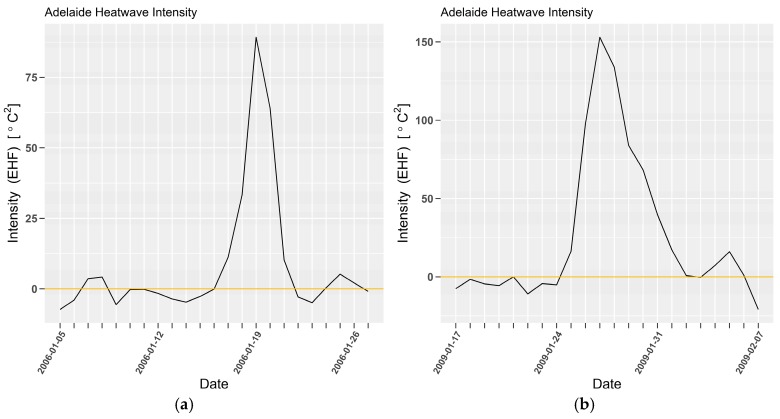
Excess Heat Factor for Adelaide’s 2006 (**a**) and 2009 (**b**) heatwaves, calculated using site data.

**Figure A6:**
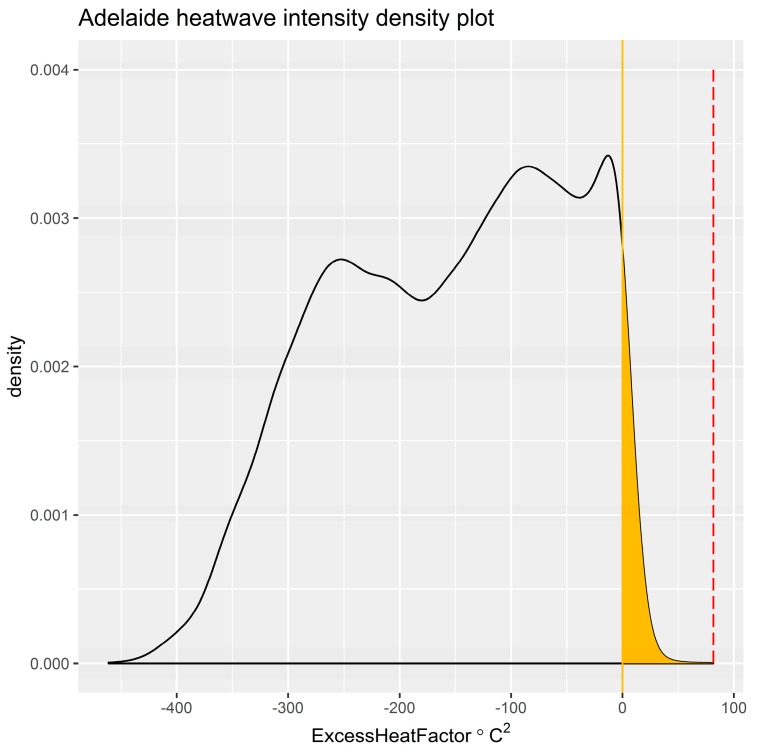
Distribution function of *EHF* for period 1887 to 2018. Values greater than zero shown in yellow are heatwaves. Maximum *EHF* value in distribution is indicated by dashed red line.

**Figure A7:**
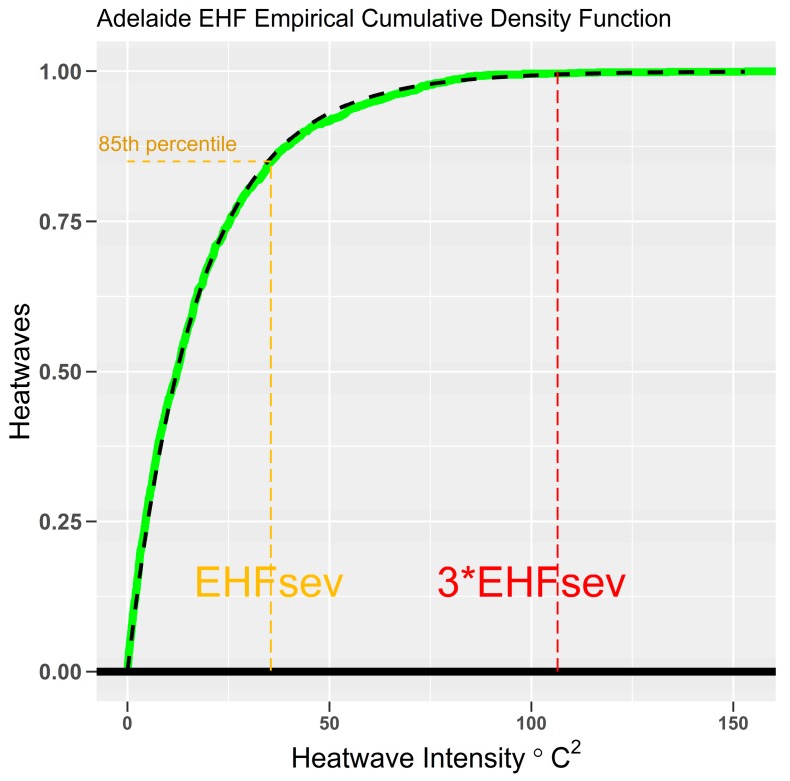
Adelaide empirical cumulative distribution of positive *EHF* (green line), overlain with the modelled generalized Pareto distribution (black dashes), and showing the 85th percentile (transition point) for determining the severe *EHF* threshold (dashed yellow lines). Extreme *EHF* threshold is shown in red (dashed red lines).

**Figure A8:**
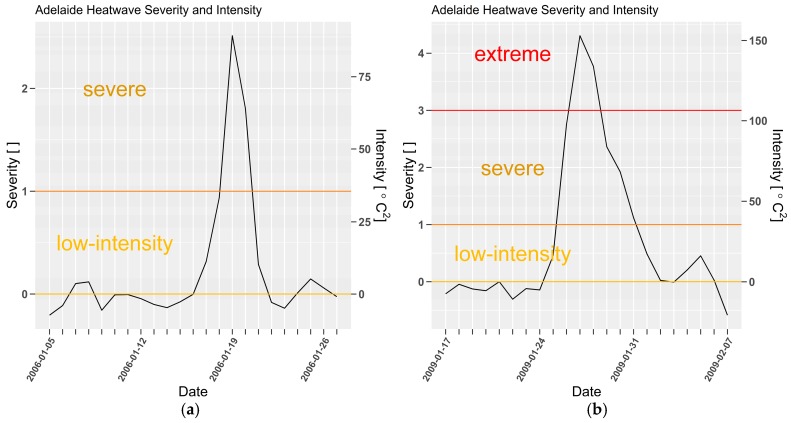
Excess Heat Factor for Adelaide’s 2006 (**a**) and 2009 (**b**) heatwaves, calculated using site data. Dimensionless heatwave severity [ ] and intensity (*EHF*, [°C^2^]) on left and right *y*-axes respectively.

## Supplementary Materials

The following are available online at http://www.mdpi.com/1660-4601/15/11/2494/s1, File S1: Heatwave Severity Moments Supplementary Materials.

## Appendix A

### Appendix A.1. Heatwave Intensity

The excess heat factor (*EHF*) [18] has been developed as a measure of heatwave intensity. The formulation of *EHF* is a factorization of two excess daily heat indices which measure:

- heat in excess of the local climatic heatwave threshold and
- heat in excess of the recent experience of heat.

These long and short-term temperature anomalies are described as the Excess Heat Index *EHI_sig_* for a significant heat event and the Excess Heat Index *EHI_accl_* for a heat event requiring an adaption or acclimatization response. These anomalies are estimated as

(A1) $$EHI_{sig}=DMT_{3-day}-DMT_{95}$$

(A2) $$EHI_{accl}=DMT_{3-day}-DMT_{30-day}$$

with *DMT*_3*-day*_ denoting the average daily temperature of the next (or t, t + 1, t + 2) 3 day period, *DMT*_95_ the percentile of daily temperatures for the 30 year reference period between 1971–2000, and *DMT*_30*-day*_ the average daily temperature of the previous 30 day period, respectively.

During a heatwave, minimum temperature significantly affects the diurnal cycle of heating. High minimum temperature will result in earlier and longer sustained high temperatures with stronger heat accumulation within the diurnal heating cycle. Consequently, the average of maximum and minimum temperatures over three days, or daily mean temperature (*DMT*), is used in these heat anomaly calculations.

Minimum and maximum temperatures are displayed in Figure A1a,b, for Adelaide heatwaves in 2006 and 2009, demonstrates the rational for using *DMT*. A reference temperature of 25 °C in these figures demonstrates the different capacity for heat to be discharged overnight. By the 8th and 9th of January 2006 the minimum has risen close to 25 °C whilst the maximums are much higher in the high 30’s. There is little capacity for the heat of the day to be discharged at night with a high minimum temperature. Consequently, this heat is accumulated with an additional heat impost the following day. Heat will not be accumulated whilst the area between the red and yellow lines (maximum temperatures and 25 °C) is balanced by the area below the yellow to the blue line (25 °C and minimum temperatures). Excess is clearly a problem by 20 January once the minimum temperature has risen above 25 °C, where there is no capacity to discharge heat.

In 2009 daily accumulations of heat are being discharged until late January, when suddenly much higher maximum and minimum temperatures commence at the same time. The much higher maximum and minimum temperatures resulted in greater excess heat producing a more intense heatwave.

The use of daily temperature (average of maximum and minimum temperatures) accommodates the concept of heat discharge.

Reference periods used for calculating Adelaide’s excess temperature anomalies are presented in Figure A2 and Figure A3. The climatological distribution of daily temperature (*DMT*) for the period 1971 to 2000 is shown in Figure A2, which illustrates the positive tail in the distribution representing all heatwave events.

Unlike the single-day, daily temperature reference period shown above, the 30-day reference period for the short-term temperature anomaly is highly subjective to the weather events occurring over that period. The climatological distribution of the acclimatization heat index utilizes 30-day running mean daily temperatures is shown in Figure A3 illustrates the sample population for the multi-week, weather determined short-term heat anomaly.

Calculation of heatwave intensity (*EHF*) is designed to treat the acclimatization index as an amplifying factor which does not reduce the significance of climate threshold excess heat;

(A3) $$EHF=EHI_{sig}\cdot \max\left(1,EHI_{accl}\right)$$

The shaded region in Figure A3 shows the distribution of warm acclimatization events that are >1. Only a sub-sample of this population subset is related to heatwaves, noting that positive 30-day anomalies can occur at any time during the year.

Adelaide heatwave examples are shown in Figure A4a,b, demonstrating the evolution of the *EHI_sig_* and *EHI_accl_* indices in 2006 and 2009.

The long and short-term temperature anomalies in 2006 show three heatwave events. *EHI_sig_* became positive for a short period in early January, with a more significant positive anomaly in both *EHI_sig_* and *EHI_accl_* lasting for a longer period. Finally, a smaller event, more significant than the first developed briefing after the major heatwave.

On two occasions during the first half of January 2009 *EHI_sig_* nearly became positive, so not heatwaves. This was followed by very large long and short-term temperature anomalies (up to 10 °C larger than those observed in 2006) resulting in an extreme heatwave that lasted for two weeks.

The same Adelaide heatwaves are shown again in Figure A5a,b, showing heatwave intensity (Excess Heat Factor).

In 2006 the intensity of the early heatwave is quite small compared to the event in the second half of January. The peak *EHF* value of 67 °C^2^ is the combination of the peak *EHI* values shown in Figure A4a. When multiplied, large *EHI_sig_* and *EHI_accl_* values will produce very large *EHF* values, indicating a very intense heatwave. Conversely, smaller *EHI*s will produce much smaller *EHF* values, indicative of low-intensity heatwaves.

The third 2006 heatwave shown in Figure A5a is of similar intensity to the first event in early January, despite the higher temperature anomalies observed in Figure A1a. This is a consequence of reduced acclimatization due to the immediately preceding intense heatwave, which can be seen in Figure A4a. It is notable that the context in which the high temperature event occurs will determine the intensity of the heatwave. The early January heatwave had low *EHI_sig_* but higher *EHI_accl_*. Figure A1a shows the lower maximum and minimum temperatures at the start of the month which contributed to the higher short-term temperature anomaly once the heatwave started. The factored *EHI*s for the third event produced the same heatwave intensity, despite the higher temperatures observed. Acclimatization (or adaptation) can either amplify or dampen the derived heatwave intensity.

In 2009 an extremely intense heatwave is shown in Figure A5a. In Figure A1b lower maximum and minimum temperatures abruptly shift into much higher values. This is evident in Figure A4b where *EHI_sig_* and *EHI_accl_* both change abruptly. The large change in acclimatization due to the much milder preceding conditions has resulted in significant amplification of the heatwave intensity signal. As seen in the 2006 example, the multiplication of two larger indices has resulted in a significant intensity signal.

*EHF* measures heatwave intensity, sensitive to the recent past (acclimatization/adaptation) and the local climate. *EHF* is location specific. The 95th percentile of daily temperature for the 1971 to 2000 climate reference period is specific to each location, and cannot be related to any other location’s climate. This is an obstacle to comparing heatwave intensity values between locations.

### Appendix A.2. Heatwave Severity

The strong signal to noise provided by factoring two temperature anomalies provides another heatwave measurement opportunity. As a quadratic calculation [°C^2^], positive *EHF* obeys a power law. The population of *EHF* shown in Figure 6A, shows the distribution of positive and negative *EHF* values. Only *EHF* values > 0 (shown in yellow) are heatwaves. The power law attributes of positive *EHF* are exhibited by the heavy tail distribution (shaded yellow) of heatwaves in this figure.

A strategy to normalize heatwave intensity has been employed, allowing direct comparison of heatwave severity, irrespective of event location.

Following Nairn and Fawcett [18], the cumulative density function of positive *EHF* (Figure A7) was demonstrated to exhibit the characteristics of a Generalized Pareto Distribution function which is suited to power function (heavy tail) distributions. It is useful to observe how the heatwave intensity (*EHF*) distribution changes in Figure A7. Low-intensity heatwaves constitute most of the heatwaves observed. In Figure A7 the 85th percentile has been highlighted with a dashed yellow line. Most, (85%) of all heatwaves are low-intensity. For the latter part of the distribution heatwave intensity rapidly becomes more intense. The transition from frequently observed, low-intensity to increasingly rare and more intense heatwaves has been identified as a transition point for population adaptation limits.

We appealed to the usefulness of the Pareto Principle [45] when considering the proximity of this transition point to the 80th percentile of the distribution function. Juran [46] observed the “vital few and trivial many”, a principle that approximately 20 percent of something are responsible for 80 percent of the results, which became known as Pareto’s Principle or the 80/20 Rule. In this application, the 85th percentile was selected as a threshold for heatwave severity as a transition point between low-intensity and severe heatwaves. Note the near perfect correspondence between empirical and modelled cumulative density in Figure A7.

A climatological record of heatwave intensity (30 years or greater) provides a stable 85th percentile *EHF* value (*EHF*_85_) which can then be used to normalize heatwave intensity into what has been called heatwave severity categories. A severity quantity of 1 at any location can be interpreted as the last 15% of the cumulative distribution heatwave days.

(A4) $$Severity=EHF÷EHF_{85}$$

Each location has a distribution of heatwave intensity that is determined by the climatological temperature range, which in turn is characterized by its own unique value of *EHF*_85_. For Adelaide, these dimensionless severity categories have been shown in Figure A8a,b, reaching category 2 in 2006 and over 4 in 2009.

Comparing heatwave intensity at a location can be achieved through analysis of heatwave intensity or severity. However, heatwaves can only be compared between locations by utilizing dimensionless severity categories.

### Appendix A.3. Summary

Daily temperature has been adopted as a means of calculating heat accumulation. High minimum temperature adjusts the rate at which high temperatures are achieved in the following diurnal cycle, and restrict the capacity for heat discharge from the previous heating cycle. In this context minimum temperature is more important than the maximum temperature in the estimation of heat accumulation.

Minimum temperature is also affected in the presence of a humid atmosphere. Water vapor is a very strong greenhouse gas, restricting the rate at which infrared radiation can escape to space, leading to higher minimum temperatures. In this heatwave intensity calculation, the resultant higher heatwave intensity incorporates the physical presence of humidity.

The use of maximum and minimum temperature data has another advantage. These parameters are available in long climate records, and are the highest quality meteorological parameters available to the community on time scales that range across climate, 7-day, multi-week, seasonal and projection forecasts. The use of a single statistically stable index across these time scales provides for forecast and warning services that are consistent with historical context and planning guidance for policy makers.

The *EHF* intensity index can be thought of in SI units as [°C^2^_L_]. The subscript _L_ has not previously been documented and is used to identify *EHF* intensity is specific to the climatology for each location where it is calculated. The stable heavy tail in the *EHF* density distribution function (Figure A6) generated by the quadratic formulation of *EHF* obeys a power law for positive *EHF*. Within this population *EHF*_85_ can be objectively determined with the same units as *EHF* at each location, [°C^2^_L_]. As a consequence, the calculation of heatwave Severity, shown in Equation (A4), is dimensionless, removing location dependency.

Dimensionless *EHF* severity is used to sensibly map heatwaves. Heatwaves may span different climatological regions and be interpreted through severity. It is notable that severity maps have been in public use in Australia since 2014. The same map of severity is used in the tropics, sub tropics and midlatitudes. Notably, regions that are normally humid during heatwaves are sensibly mapped using severity maps.

The creation of a dimensionless heatwave severity index allows the comparison of heatwaves irrespective of location. Historical, current, or forecast events can be compared to consider the scale of the physical, sensible heat impost. Impacts across infrastructure, utilities, human health, and social assets are exposed and their response measured.
